# The association between insight and depressive symptoms in schizophrenia: Undirected and Bayesian network analyses

**DOI:** 10.1192/j.eurpsy.2020.45

**Published:** 2020-05-06

**Authors:** Mario Amore, Martino Belvederi Murri, Pietro Calcagno, Paola Rocca, Alessandro Rossi, Eugenio Aguglia, Antonello Bellomo, Giuseppe Blasi, Bernardo Carpiniello, Alessandro Cuomo, Liliana dell’Osso, Massimo di Giannantonio, Giulia Maria Giordano, Carlo Marchesi, Palmiero Monteleone, Cristiana Montemagni, Lucio Oldani, Maurizio Pompili, Rita Roncone, Rodolfo Rossi, Alberto Siracusano, Antonio Vita, Patrizia Zeppegno, Alessandro Corso, Costanza Arzani, Silvana Galderisi, Mario Maj

**Affiliations:** 1 Section of Psychiatry, Department of Neurosciences, Rehabilitation, Ophthalmology, Genetics sand Maternal and Child Health, University of Genoa, Genoa, Italy; 2 Department of Neuroscience, Section of Psychiatry, University of Turin, Turin, Italy; 3 Section of Psychiatry, Department of Biotechnological and Applied Clinical Sciences, University of L’Aquila, L’Aquila, Italy; 4 Department of Clinical and Molecular Biomedicine, Psychiatry Unit, University of Catania, Catania, Italy; 5 Psychiatry Unit, Department of Medical Sciences, University of Foggia, Foggia, Italy; 6 Department of Neurological and Psychiatric Sciences, University of Bari, Bari, Italy; 7 Section of Psychiatry, Department of Public Health, Clinical and Molecular Medicine, University of Cagliari, Cagliari, Italy; 8 Department of Molecular Medicine, University of Siena, Siena, Italy; 9 Section of Psychiatry, Department of Clinical and Experimental Medicine, University of Pisa, Pisa, Italy; 10 Department of Neuroscience and Imaging, G. D’Annunzio University, Chieti, Italy; 11 Department of Psychiatry, University of Campania “Luigi Vanvitelli”, Naples, Italy; 12 Department of Neuroscience, Psychiatry Unit, University of Parma, Parma, Italy; 13 Department of Medicine, Surgery and Dentistry “Scuola Medica Salernitana” Section of Neuroscience, University of Salerno, Salerno, Italy; 14 Department of Psychiatry, University of Milan, Milan, Italy; 15 Department of Neurosciences, Mental Health and Sensory Organs, S. Andrea Hospital, Sapienza University of Rome, Rome, Italy; 16 Unit of Psychiatry, Department of Life, Health and Environmental Sciences, University of L’Aquila, L’Aquila, Italy; 17 Department of Systems Medicine, Psychiatry and Clinical Psychology Unit, Tor Vergata University of Rome, Rome, Italy; 18 Psychiatric Unit, School of Medicine, University of Brescia, Brescia, Italy; 19 Department of Mental Health, Spedali Civili Hospital, Brescia, Italy; 20 Department of Translational Medicine, Psychiatric Unit, University of Eastern Piedmont, Novara, Italy; 21 Department of Biomedical and Specialty Surgical Sciences, Institute of Psychiatry, University of Ferrara, Ferrara, Italy

**Keywords:** Demoralization, depression, insight, sadness, schizophrenia, self-esteem

## Abstract

**Background.:**

Greater levels of insight may be linked with depressive symptoms among patients with schizophrenia, however, it would be useful to characterize this association at symptom-level, in order to inform research on interventions.

**Methods.:**

Data on depressive symptoms (Calgary Depression Scale for Schizophrenia) and insight (G12 item from the Positive and Negative Syndrome Scale) were obtained from 921 community-dwelling, clinically-stable individuals with a DSM-IV diagnosis of schizophrenia, recruited in a nationwide multicenter study. Network analysis was used to explore the most relevant connections between insight and depressive symptoms, including potential confounders in the model (neurocognitive and social-cognitive functioning, positive, negative and disorganization symptoms, extrapyramidal symptoms, hostility, internalized stigma, and perceived discrimination). Bayesian network analysis was used to estimate a directed acyclic graph (DAG) while investigating the most likely direction of the putative causal association between insight and depression.

**Results.:**

After adjusting for confounders, better levels of insight were associated with greater self-depreciation, pathological guilt, morning depression and suicidal ideation. No difference in global network structure was detected for socioeconomic status, service engagement or illness severity. The DAG confirmed the presence of an association between greater insight and self-depreciation, suggesting the more probable causal direction was from insight to depressive symptoms.

**Conclusions.:**

In schizophrenia, better levels of insight may cause self-depreciation and, possibly, other depressive symptoms. Person-centered and narrative psychotherapeutic approaches may be particularly fit to improve patient insight without dampening self-esteem.

## Highlights

- Better insight seems associated with depressive symptoms in schizophrenia.
- Network analyses were used to explore this association in a large sample.
- Insight was associated with self-depreciation, guilt, and suicidal ideation.
- Although cross-sectional, data suggest causal direction from insight to depression.

## Introduction

Better levels of insight are associated with the presence of depressive symptoms among patients with schizophrenia, but it would be useful to understand the relationship at symptom level.

Lack of awareness into the illness is a common feature of schizophrenia that hampers adherence to treatment and complicates the clinical course [1, 2]. Conversely, having good levels of insight may also bring about negative consequences, namely depressive symptoms [3] or even suicidal ideation [4, 5]. This phenomenon has been termed the “insight paradox” and possibly results from the painful realization of the implications and consequences of suffering from a chronic and stigmatized illness. The association between good insight and depression was examined by different individual studies assessing patients at different stages of the disorder [3, 4, 6–10]. Pooled results suggest that this phenomenon is complex and highly variable, both in terms of strength of the association and underlying psychopathological mechanisms [11]. Moreover, this relationship may depend on a number of clinical, contextual, and cultural factors, such as socioeconomic status, engagement with mental health services, stigmatization, and severity of the illness [1, 6, 12, 13]. Moreover, several potential confounders can be identified, for example, negative and positive symptoms [14], hostility [15], cognitive abilities [16, 17], levels of social cognition [18], and extrapyramidal side effects [19].

Besides clinical and sociodemographic variability, previous inconsistent findings could be also related to methodological factors. Several studies examining the relationship between insight and depression examined relatively small sample sizes, and only few measured multiple confounders at once, thus possibly preventing the detection of significant associations. To this end, the network approach to psychopathology may offer a suitable approach to examine the associations linking insight to specific depressive symptoms [20].

Given these premises, the aim of this study was to examine the relationship between insight and depressive symptoms in a large representative sample of patients with schizophrenia, using the network approach to psychopathology. We hypothesized that insight would be associated with self-depreciation and suicidal ideation and that the most likely causal direction would be from insight to depressive symptoms.

## Methods

### Study population

This is a reanalysis of data collected in the study by the Italian Network for Research on Psychoses: a detailed description of the study procedures is provided elsewhere [21]. Briefly, a large representative sample of clinically stable, community-dwelling patients aged 18–66 with a diagnosis of schizophrenia were recruited from various outpatient units of 26 Italian university psychiatric clinics and mental health departments. Clinical stability was defined as the absence of variation of antipsychotic drug treatment and of hospitalizations during the 3 months before recruitment [21]. Exclusion criteria were: presence of neurological disorders; history of alcohol dependence or substance abuse in the past 6 months; moderate or severe mental retardation; and inability to provide informed consent. The study was approved by the local ethics committees and was conducted in accordance with the Helsinki Declaration as revised in 1989.

### Assessments

The study involved the collection of detailed sociodemographic and clinical information. Briefly, the severity of depressive symptoms was assessed with the Calgary Depression Scale for Schizophrenia (CDSS) [22]. The scale comprises nine items, namely depression, hopelessness, self-depreciation, guilty ideas of reference, pathological guilt, morning depression, early wakening, suicide, and observed depression. Insight was assessed using the G12 item of the Positive and Negative Syndrome Scale (PANSS) [23]. The item rates the severity of “lack of judgment and insight,” with higher scores indicating lower levels of insight. Hostility was rated with the item P7, where higher scores indicate greater hostility. PANSS factor scores for the dimensions “disorganization” and “positive symptoms” were calculated according to the 5-factor solution by Wallwork et al. [24]. The severity of negative symptoms was assessed using the Brief Negative Symptom Scale (BNSS) and calculating scores for the “poor emotional expression” and “avolition” factors [25]. Extrapyramidal symptoms were assessed with the St. Hans Rating Scale (SHRS) total score, converted in *z*-scores; higher scores indicate more severe extrapyramidal symptoms [26]. Neurocognitive functions were assessed using the measurement and treatment research to improve cognition in schizophrenia (MATRICS) consensus cognitive battery (MCCB) [27]. The composite score was calculated excluding the Mayer–Salovey–Caruso Emotional Intelligence Test (MSCEIT) score and adjusting for age and gender. Social cognition was assessed with the “managing emotion” section of the MSCEIT, the Facial Emotion Identification Test (FEIT) [28] and the Awareness of Social Inference Test (TASIT) [29]. Data was reduced to two factors with principal component analysis, with greater scores indicating better social cognition abilities. Internalized stigma was assessed with the Internalized Stigma of Mental Illness (ISMI) total score: higher scores indicate greater levels of internalized stigma [30]. The Perceived Devaluation and Discrimination Scale (PDD) [31] was used to assess perceived discrimination, again, higher scores indicate greater perceived discrimination. The Service Engagement Scale measured the levels of engagement with mental health services; higher scores indicating worse levels of engagement with services [32]. Socioeconomic status was estimated with the Hollingshead Index (HI) [33].

### Data analyses

Depression is usually assessed by means of rating scale sum scores, typically, the Calgary Depression Scale for Schizophrenia [19]. Despite its utility, this approach prevents from detecting potential associations between contextual or clinical features and specific depressive symptoms, which could represent elective targets for psychological [34–36] or pharmacological [37] interventions against depression in schizophrenia. Whereas, network analyses provide information on the relationship between individual symptoms of mental disorders by modeling their mutual relationship [20]. Network analyses allow to depict the interactions between individual symptoms representing them as “nodes” that are interconnected by “edges,” the latter representing the strength of symptoms’ mutual interactions [38].

In the first analysis, we examined the network of depressive symptoms comprising items from the CDSS and insight, rated by the PANSS item G12. The network was estimated using the *qgraph* 1.6.5 package, with EBICglasso regularization [39]. The procedure relies on the selection of the most meaningful partial correlations between individual symptoms, represented as edges of varying thickness. The network is visualized using the Fruchterman–Reingold algorithm, which positions nodes with more relevant connections more centrally in the network. We report on the network centrality measure of node strength, that is the sum of the weights of all direct connections between a specific symptom and the others in the network. Also, we highlight the shortest paths linking the node of insight to those of depressive symptoms. Network accuracy was estimated with: (a) estimation of the bootstrapped confidence intervals of edge-weights from a nonparametric bootstrap procedure (*n* = 1,000); (b) estimation of the stability of node centrality (strength index) from a case-drop bootstrap procedure (*n* = 1,000). Here, an increasing proportion of cases is subtracted from the dataset, while re-estimating the network structure and centrality indices multiple times. Node strength stability is represented graphically and indexed by the Correlation Stability Coefficient (CS-C), that is the maximum proportion of cases that can be dropped from the sample with minimal impact on centrality indices. These procedures are implemented in the *bootnet* 1.3 package [39].

Second, we examined the effect of previously identified moderators (i.e., effect modifiers) on the network of insight and depressive symptoms [6]. The sample was subdivided based on a median split of each moderating variable (HI scores for socioeconomic status, SES scores for engagement with mental health services, and PANSS total scores for illness severity) and the structures of the resulting networks were compared. The package NetworkComparisonTest 2.2.1 provides information on the differences in global network structure, global network strength and strength of individual edges, based on a permutation test (*n* = 1,000) [40].

Third, we examined whether the relationship between insight and depressive symptoms would change after including confounders and putative mediators in the model. To this end, we re-estimated the network structure after adding the following factors: PANSS factors for disorganized and positive symptoms, PANSS item p7 for hostility, BNSS factors for negative symptoms, neurocognitive and social cognition factors, ISMI total score for internalized stigma, PDD score for perceived discrimination, and SHRS score for the severity of extrapyramidal symptoms.

Fourth, we sought to determine the putative direction of the causal relationship between insight and depression by reanalyzing data of depressive symptoms and insight with a directed acyclic graph (DAG). This is a recently developed addition to network analyses based on a Bayesian approach. Unlike undirected networks, Bayesian DAGs have been developed to detect and represent the most likely direction of the *causal* relationships between symptoms based on the conditional dependence between each couple of variables, given the other variables in the network [41, 42]. For this purpose, the package *bnlearn* 4.5 was used [43], following the procedures described in a recent paper [42]. Briefly, the procedure “learns” the structure of the network using a hill-climbing algorithm that computes the structure of the directed network multiple times (*n* = 1,000 iterations) while computing a goodness-of-fit index (i.e., Bayesian Information Criteria, BIC) for each edge. By relying on a validated threshold-based method to optimize sensitivity and specificity [44], a final, averaged network is selected. The network retains only those edges and causal directions that appear in a substantial proportion of the iterations, defined by the threshold. For ease of interpretation, only those edges displaying the same direction in 70% or more of the iterations were plotted as directed (i.e., with arrows), while the remainder were plotted as undirected. Moreover, the averaged network was plotted in two ways: (a) weighting the thickness of each edge by its fitted regression parameter, in order to display the magnitude and direction of the connections between nodes and (b) weighting the thickness of each edge by its arc strength, thus indicating the relative importance of each edge in the network. Arc strength indicate the magnitude of the corresponding BIC value for each edge; hence, a large negative value for one edge suggests that removing it would significantly worsen the network fitness.

All analyses were carried out in R version 3.6.1 The R code and correlation matrices are available in the supplement for ease of reproducibility.

## Results

### Network of insight and depressive symptoms

**Figure 1.**
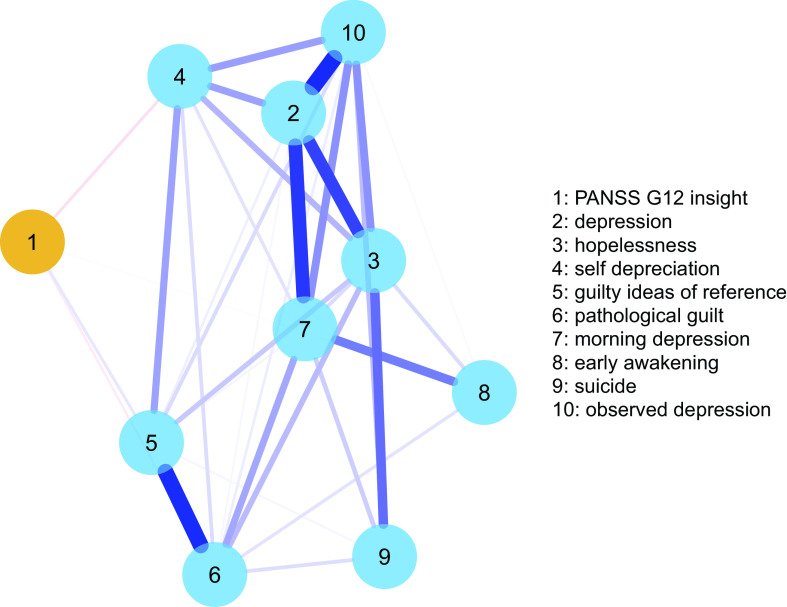

Table 1 reports the characteristics and rating scale scores of the 921 participants in the study. Three participants had missing data for insight or depression scales. The network of depressive symptoms and insight contained 35 edges (Figure 1, weighted adjacency matrix in Table S1). Depressive symptoms were highly interconnected, and lack of insight was connected negatively with (in decreasing order of edge weight) self-depreciation, guilty ideas of reference, pathological guilt, and positively with early wakening. This indicates that greater levels of insight were associated with more severe depressive symptoms, with the exception of early wakening. Lack of insight was the least central node in the network, while depressed mood and hopelessness were the most central (centrality plot is reported in Figure S1). The shortest paths between insight and depressive symptoms are reported in Table S2. The link between insight and self-depreciation was the shortest path to hopelessness, suicide and observed depression. The network appeared quite stable across bootstrap accuracy analyses, both in terms of edge weights and node strength (Figures S2 and S3). The case-drop procedure indicated that 80% of the sample could be dropped without significantly affecting node strength.

**Table 1.** Sample characteristics (*n* = 921).

Sociodemographic and clinical features	
Gender (% males)	69.6
Age (years, mean ± SD)	40.2 ± 10.7
Married (% yes)	7.8
Working (%yes)	29.2
Education (years, mean ± SD)	11.6 ± 3.4
Hollingshead index (mean ± SD)	25.6 ± 14.8
Age at first psychotic episode (years, mean ± SD)	24.0 ± 7.2
Antipsychotic treatment (%yes)	
First generation	14.2
Second generation	48.5
Both	14.1
None	3.2
Suicide attempts (% yes)	17.1
Rating scales scores (mean ± SD)	
CDSS total score	4.0 ± 4.0
PANSS total score	75.5 ± 23.0
PANSS positive factor	9.8 ± 4.7
PANSS disorganization factor	8.56 ± 3.80
PANSS item G12	3.18 ± 1.62
PANSS item P7	1.71 ± 1.11
BNSS poor emotional expressivity	12.8 ± 8.0
BNSS avolition	20.7 ± 9.6
Neurocognitive component	26.7 ± 11.9
Social cognition factor 1	−1.31 ± 1.09
Social cognition factor 2	−1.55 ± 1.26
SES	12.9 ± 7.7
ISMI	2.1 ± 0.5
PDD	2.57 ± 0.50
St. Hans rating scale (*z*-scores, median, range)	−0.39 (−0.44–5.47)

Abbreviations: BNSS, Brief Negative Symptom Scale; CDSS, Calgary Depression Scale for Schizophrenia; ISMI, Internalized Stigma of Mental Illness; PDD, Perceived Devaluation and Discrimination Scale; PANSS, Positive and Negative Syndrome Scale; SD, standard deviation.

### Role of moderators

Socioeconomic status, service engagement and illness severity were weakly correlated with nodes in the network (Table S3). We compared the networks of insight and depressive symptoms between subgroups of participants subdivided by different levels of the three putative moderators. There were no significant differences in network characteristics between participants with low versus high socioeconomic status (406 versus 384 subjects, respectively; network invariance test *M* = 0.17, *p* = 0.25; global strength invariance test *S* = 0.02, *p* = 0.93, Figures S4–S6) or subjects with low versus high engagement with mental health services was (453 vs. 465 subjects, respectively, network invariance test *M* = 0.14, *p* = 0.42; global strength invariance test: low SES: 3.18, high SES: 3.64, *S* = 0.47, *p* = 0.06, Figures S7–S9). There were no significant differences in edges linking insight with depressive symptoms (all *p* > 0.05). Lastly, there were no significant global network differences comparing the networks of individuals with low versus high illness severity (451 vs. 458, respectively, network invariance test *M* = 0.18, *p* = 0.07; global strength invariance test *S* = 0.30, *p* = 0.20, Figures S10–S12). However, among individuals with low severity, insight was negatively connected with hopelessness (higher insight/greater hopelessness, edge weight = −0.027) and pathological guilt (edge weight = −0.10), while among individuals with higher severity insight did not display connections with these nodes (*p* = 0.04 and *p* = 0.007, respectively).

### Extended network

**Figure 2.**
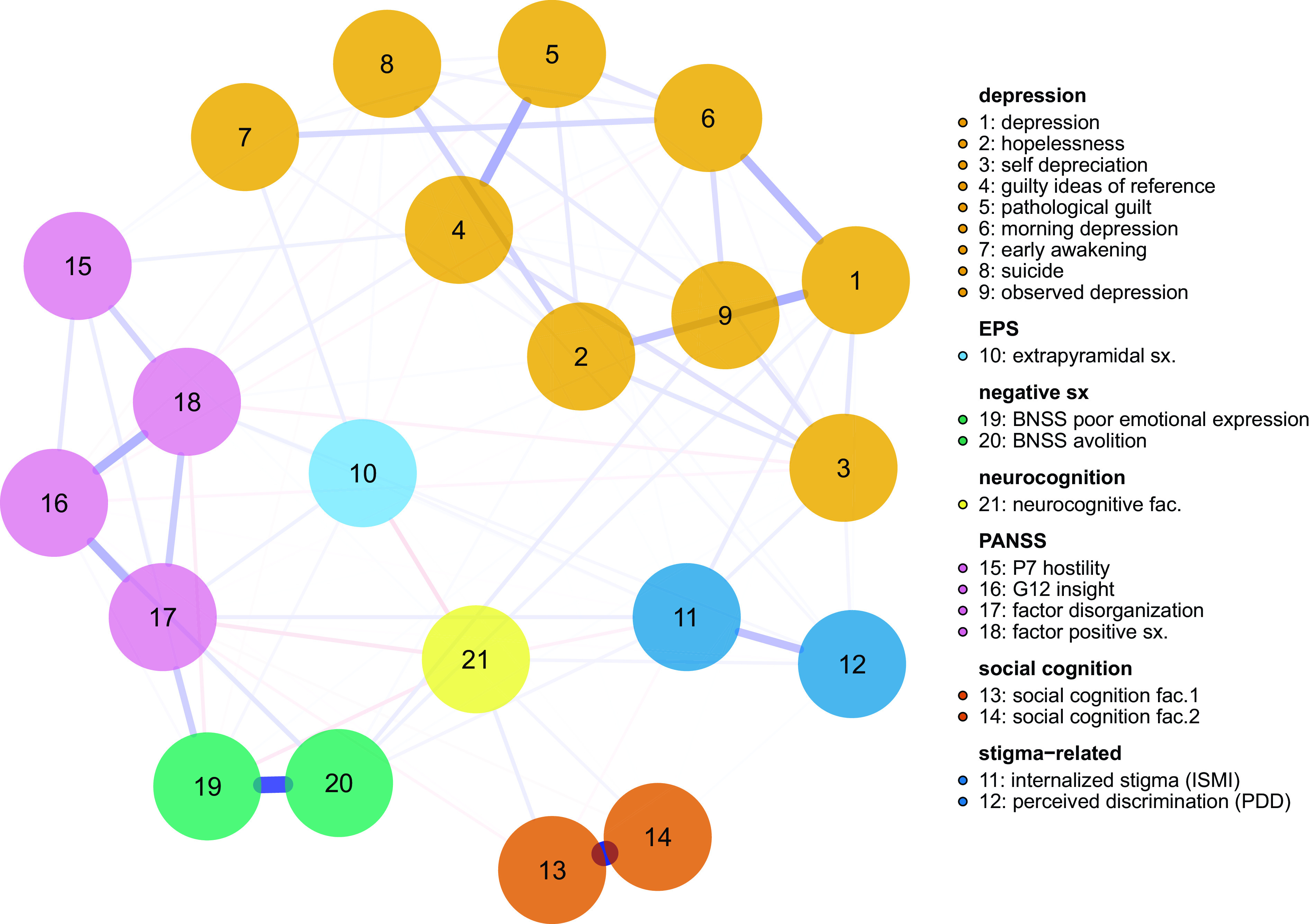

After entering confounding factors in the network (Figure 2) insight displayed negative edges with self-depreciation, morning depression and pathological guilt, similar to the previous network, and, in addition, with suicide (greater the levels of insight/more severe depressive symptoms). Lack of insight was also connected by positive edges with hostility, disorganization and positive symptoms (PANSS factors) and negative symptoms (BNSS factors), while it displayed negligible or no connections with internalized stigma (ISMI), perceived discrimination (PDD), social cognition and neurocognition. Hostility was also positively connected with some depressive symptoms (guilty ideas of reference, early wakening, suicide). Results of centrality analyses and network accuracy bootstrap analyses are reported in the supplement (Figures S13–S15).

### Directed acyclic graph

**Figure 3.**
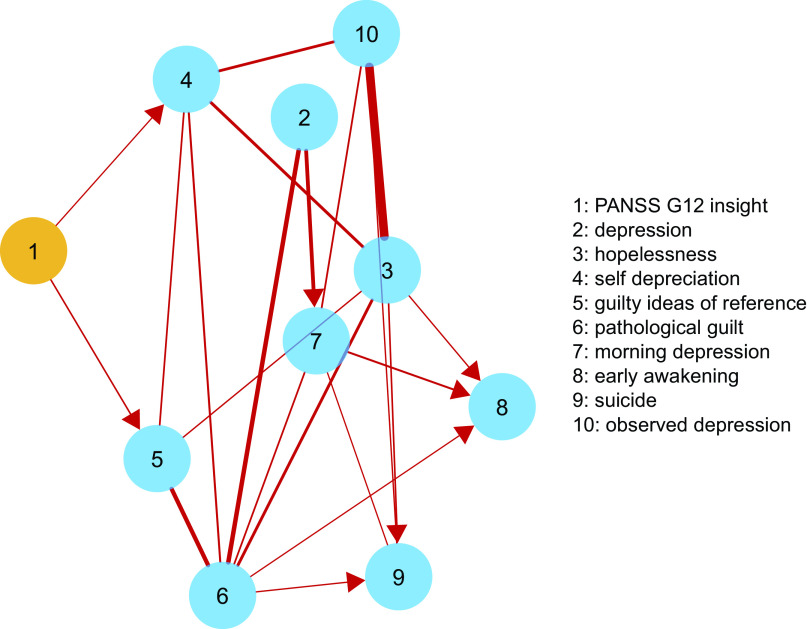

The procedure by [44] identified 0.466 as the optimal significance threshold. Thus, edges appearing in 46.6% or more, of the bootstrapped networks were retained in the averaged DAG of insight and depressive symptoms; this corresponded to 21 edges (Figure 3). In the DAGs (Figure 3) only those edges with an unequivocal direction (70% or more iterations displaying the same direction) were plotted as directed. The percentage of bootstrap iterations reporting the same direction for each edge is reported in Table S4.

Higher levels of insight predicted higher levels of self-depreciation (negative coefficient: −0.21) and lower levels of guilty ideas of reference (positive coefficient: 0.06). The most likely direction of their causal relationship was from insight to depressive symptoms (self-depreciation: 96%; guilty ideas of reference: 89%). The rest of connections between depressive symptoms were all positive, those with greater magnitude being from pathological guilt to early awakening, from morning depression to suicide, from insight to self-depreciation and from pathological guilt to self-depreciation (Figure 3 and Table S4). The most important edges in terms of arc strengths (fitness of the network model to data), were from hopelessness to observed depression, from pathological guilt to depression, from pathological guilt to guilty ideas of reference and from depression to morning depression (Figure S16 and Table S4).

## Discussion

The study examined the relationship between insight and depressive symptoms in schizophrenia, a clinical phenomenon that has been termed “insight paradox.” Having good insight is generally regarded as a favorable clinical feature: nonetheless, patients with greater awareness into their illness may present with depressive symptoms—a seemingly contradictory finding [8]. By relying on the network approach to psychopathology and examining a large, representative clinical sample of clinically stable patients, we highlighted the associations between insight and specific depressive symptoms, while accounting for the role of moderators and confounding factors.

The main finding is that good levels of insight were associated with greater self-depreciation, as well as pathological guilt, morning depression and suicide. The finding is in line with results from the meta-analysis on this topic [11] and our previous study [6], although we did not replicate the effects of illness severity, socioeconomic status and service engagement [6]. These results, however, extend previous findings by identifying which depressive symptoms could derive from the acquisition of insight and detecting the most probable direction of their causal relationship.

Results from the DAG of insight and depression are not directly comparable with those from the undirected networks: among other differences, DAGs cannot include feedback or feedforward loops, and they are estimated differently [44]. Nonetheless, both analyses identified a connection between increased insight and self-depreciation, with the most likely direction going from insight to self-depreciation, rather than vice versa. These results are in keeping with findings from prospective studies: improvements of insight predicted the future development of depressive symptoms in the majority of works examining this issue [7, 45–49], while only one detected a reciprocal putative causal direction [50]. These findings have been interpreted according to a “defensive” role of low insight, which would protect the individual against self-devaluation and low mood [11, 51, 52]. Importantly, the inclusion of other symptom dimensions as confounders in our model did not affect the strength and direction of such associations but, on the contrary led to detect an additional connection with suicidal ideation. Thus, in order to detect reliable associations between insight and depression, other symptom dimensions and clinical features should be taken in account [14, 16, 53, 54].

The association of better insight with self-depreciation may reflect the dire psychological consequences of the subjective illness experience: several studies have shown that greater awareness of one’s own mental illness can dampen self-esteem [8, 55–57] by evoking a wide array of negative feelings: shame, loss, subjective doubt, perceived burdensomeness, and guilt [58–62]. The degree of identification with one’s illness (i.e., patient role) becomes particularly problematic in the presence of stigma, either endorsed by patients [63, 64] or by their relatives [65]. These dynamics may pave the way to demoralization [66, 67], hopelessness and suicidal ideation [4, 68, 69], unless individuals are helped to come to terms with, and make sense of their illness. To this end, a person-centered approach, focused on the development of metacognitive abilities and relying on a narrative approach has yielded promising results for the improvement of insight without lowering self-esteem [36, 70].

Finally, greater levels of insight were associated (in the adjusted network, but not in the DAG) with higher morning depression, a common sign of sleep disruption and/or neuroendocrine circadian abnormalities across various psychiatric disorders [71]. In this light, it should be recognized that not only psychosocial, but also biological mechanisms may underlie the relationship between insight and specific depressive symptoms, as well as between other clinical dimensions. Future studies are still needed to elucidate the differential mechanisms of symptom–symptom interaction.

This study is strengthened by a large, representative sample of individuals suffering from schizophrenia and the use of state-of-the art analytic techniques that are fit to describe complex dynamic systems, such as psychopathology [72]. However, our findings need to be weighed in the light of the study limitations. First, the PANSS item used to rate insight is less detailed than multidimensional tools [2]. Using the PANSS item instead of other instruments prevents from making inferences on which sub-dimensions of insight are linked with depressive symptoms (i.e., awareness of the illness, of social consequences or perceived treatment need). Moreover, the PANSS item rates “lack of judgment” besides insight [23]: this relative lack of specificity may have led to underestimate, or confound the magnitude of the association with depressive symptoms. Future studies should encompass multidimensional assessment tools in order to provide more specific information that may guide psychoeducational or psychotherapeutic interventions. Second, even if the DAG provides an indication on the most likely causal direction of the symptom interactions, it is based on cross-sectional data, calling for caution when interpreting this finding. Whereas, future longitudinal network analyses may be particularly suited to re-examine this issue, possibly modeling the short-term interactions between insight and depressive symptoms [73]. Third, although we assessed a wide range of clinical dimensions, we did not assess specific psychological features that are likely to play a role in the association between insight and depression, such as shame, self-esteem, or illness-related appraisals. Their inclusion in the model may further contribute to map the dynamics at play. Fourth, comparing the network for different levels of illness severity might have introduced Berkson’s bias [74]: thus, additional edges, in particular of negative sign, must be interpreted with caution. Future studies on this issue should also take advantage of a recently-introduced approach, namely moderated network models [75].

In conclusion, greater levels of insight can lead to the development of self-reproach, guilt or even suicidal ideation among patients with schizophrenia. Clinicians should be aware of the delicacy of discussing issues related to diagnostic and prognostic issues with patients, while actively investigating self-esteem, stigmatization and suicidal ideation among individuals with preserved insight [4].

## Data Availability

The code is available in the supplementary online materials. Data that support the findings of this study are not available.

## Supplementary material

For supplementary material accompanying this paper visit http://dx.doi.org/10.1192/j.eurpsy.2020.45. click here to view supplementary material

## Appendix

### The members of the Italian Network for Research on Psychoses involved in this study

Members of the Italian Network for Research on Psychoses involved in this study include: Giulia Petrilli and Matteo Respino (University of Genoa); Marco Papalino, Andrea Falsetti, and Vita Maria Calia (University of Bari); Stefano Barlati, Giacomo Deste, and Cesare Turrina (University of Brescia); Federica Pinna, Alice Lai, and Silvia Lostia di Santa Sofia (University of Cagliari); Maria Salvina Signorelli and Antonio Petralia (University of Catania); Mauro Pettorruso, Gaia Barone, and Anatolia Salone (University of Chieti); Giuseppe Piegari, Carmen Aiello, Francesco Brando, and Luigi Giuliani (University of Campania “Luigi Vanvitelli,” Naples); Mario Altamura, Raffaella Carnevale, and Flavia Padalino (University of Foggia); Laura Giusti, Anna Salza, Donatella Ussorio, Giulia Pizziconi, Valeria Santarelli, and Francesca Pacitti (University of L’Aquila); Andrea de Bartolomeis (University of Naples Federico II); Eleonora Gambaro, Eleonora Gattoni, and Carla Gramaglia (University of Eastern Piedmont, Novara); Chiara De Panfilis, Paolo Ossola, and Matteo Tonna (University of Parma); Claudia Carmassi, Barbara Carpita, and Ivan Cremone (University of Pisa); Anna Comparelli, Valentina Corigliano, and Roberto Brugnoli (Sapienza University of Rome); Giulio Corrivetti, Giammarco Cascino, and Gianfranco Del Buono (Department of Mental Health, Salerno); Andrea Fagiolini, Simone Bolognesi, and Arianna Goracci (University of Siena); Giorgio Di Lorenzo, Cinzia Niolu, and Michele Ribolsi (Tor Vergata University of Rome); Silvio Bellino, Paola Bozzatello, and Claudio Brasso (University of Turin).
