# Supplementary material for: The association between insight and depressive symptoms in schizophrenia: Undirected and Bayesian network analyses
Source: Eur Psychiatry. 2020 May 6;63(1):e46. doi: 10.1192/j.eurpsy.2020.45 (PMC7358633; doi:10.1192/j.eurpsy.2020.45)
Supplement: Supplementary file 1 [file S0924933820000450sup001.docx]

**Figure S1. Centrality plot for the network of depressive symptoms and insight**

**Table S1. Weighted adjacency matrix for the network of depressive symptoms and insight**

|  | 1. G12_insight | 2. depression | 3. hopelessness | 4. self_deprec | 5. guilty_id_refer | 6. pathol_guilt | 7. morning_depr | 8. early_wake | 9. suicide | 10. observ_depres |
| --- | --- | --- | --- | --- | --- | --- | --- | --- | --- | --- |
| G12_insight | - | - | - | -0.04 | 0.04 | -0.03 | - | 0.01 | - | - |
| depression | - | - | 0.25 | 0.13 | 0.03 | 0.02 | 0.28 | - | - | 0.34 |
| hopelessness | - | 0.25 | - | 0.11 | 0.08 | 0.10 | 0.06 | 0.06 | 0.19 | 0.15 |
| self_deprec | -0.04 | 0.13 | 0.11 | - | 0.13 | 0.06 | 0.05 | - | - | 0.14 |
| guilty_id_refer | 0.04 | 0.03 | 0.08 | 0.13 | - | 0.33 | 0.02 | - | 0.02 | 0.06 |
| pathol_guilt | -0.03 | 0.02 | 0.10 | 0.06 | 0.33 | - | 0.13 | 0.05 | 0.06 | 0.03 |
| morning_depr | - | 0.28 | 0.06 | 0.05 | 0.02 | 0.13 | - | 0.17 | 0.08 | 0.15 |
| early_wake | 0.01 | - | 0.06 | - | - | 0.05 | 0.17 | - | - | 0.01 |
| suicide | - | - | 0.19 | - | 0.02 | 0.06 | 0.08 | - | - | 0.10 |
| observ_depres | - | 0.34 | 0.15 | 0.14 | 0.06 | 0.03 | 0.15 | 0.01 | 0.10 | - |

**Table S2. Shortest paths from insight to depressive symptoms in the undirected network**

| **Origin node** | **Destination node** | **Shortest path** |
| --- | --- | --- |
| insight | depression | self_deprec - depression |
| insight | hopelessness | self_deprec - hopelessness |
| insight | self_deprec | self_deprec |
| insight | guilty_id_refer | guilty_id_refer |
| insight | pathol_guilt | guilty_id_refer - pathol_guilt |
| insight | morning_depr | guilty_id_refer - pathol_guilt - morning_depr |
| insight | early_wake | guilty_id_refer - pathol_guilt - morning_depr - early_wake |
| insight | suicide | self_deprec – hopelessness - suicide |
| insight | observ_depres | self_deprec - observ_depres |

**Figure S2. Edge-weight accuracy estimated by bootstrapped 95% confidence intervals**

**Figure S3. Node strength stability in the network of depressive symptoms and insight: case-dropping bootstrap procedure**

Maximum drop proportions to retain correlation of 0.7 in at least 95% of the samples (CS-coefficient): edge: 0.80; strength: 0.80

**Table S3. Correlations between putative moderators and nodes in the network**

|  | **panss_g12** | **CDSS_total** | **depression** | **hopelessness** | **self_deprec** | **guilty_id_refer** | **pathol_guilt** | **morning_depr** | **early_wake** | **suicide** | **observ_depres** |
| --- | --- | --- | --- | --- | --- | --- | --- | --- | --- | --- | --- |
| **PANSS_total** | **,652^**^** | **,340^**^** | **,269^**^** | **,278^**^** | **,201^**^** | **,300^**^** | **,202^**^** | **,185^**^** | **,153^**^** | **,135^**^** | **,271^**^** |
|  | ,000 | ,000 | ,000 | ,000 | ,000 | ,000 | ,000 | ,000 | ,000 | ,000 | ,000 |
|  | 920 | 918 | 918 | 918 | 918 | 918 | 918 | 918 | 918 | 918 | 918 |
| **HI** | **-,052** | **,111^**^** | **,105^**^** | **,114^**^** | **,082^*^** | **,069** | **,045** | **,074^*^** | **-,003** | **,060** | **,101^**^** |
|  | ,147 | ,002 | ,003 | ,001 | ,022 | ,052 | ,203 | ,038 | ,943 | ,090 | ,005 |
|  | 792 | 790 | 790 | 790 | 790 | 790 | 790 | 790 | 790 | 790 | 790 |
| **SES_Tot** | **,310^**^** | **,059** | **,037** | **,049** | **,004** | **,044** | **-,007** | **,026** | **,108^**^** | **,070^*^** | **,033** |
|  | ,000 | ,075 | ,261 | ,139 | ,915 | ,178 | ,827 | ,425 | ,001 | ,035 | ,325 |
|  | 920 | 918 | 918 | 918 | 918 | 918 | 918 | 918 | 918 | 918 | 918 |

**Pearson’s R; ** p<0.01**

**Figure S4. Comparison by socioeconomic status (Hollingshead index)**

| **Subgroup** | **n** | **Mean HI (sd)** | **CS-coefficient edge** | **CS-coefficient node strength** |
| --- | --- | --- | --- | --- |
| Low HI | 406 | 13.87 (6.27) | 0.672 | 0.672 |
| High HI | 384 | 37.90 (10.55) | 0.672 | 0.750 |

CS-coefficients: maximum drop proportions to retain correlation of 0.7 in at least 95% of the samples


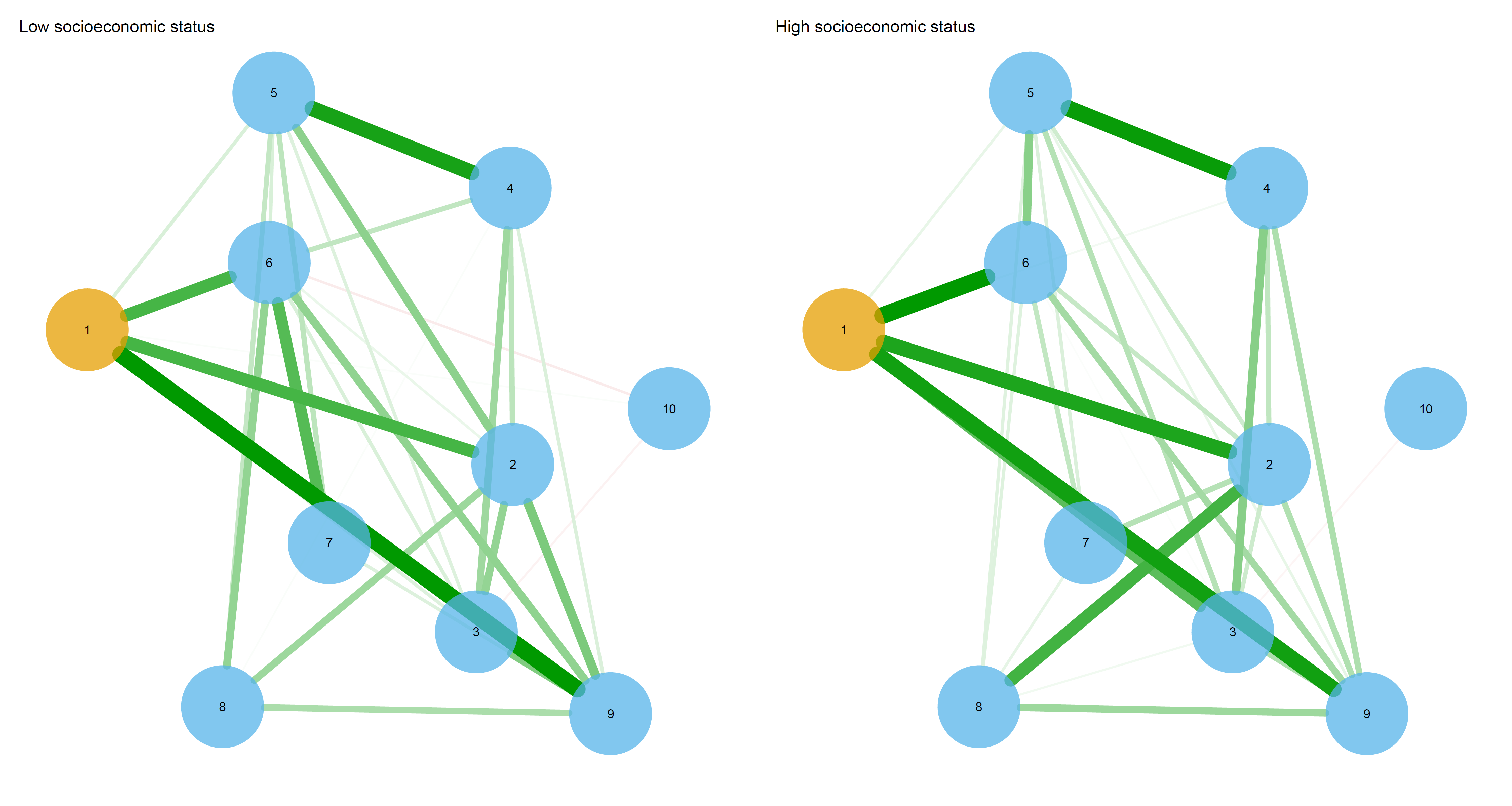


**Legend.** 1: PANSS G12 insight; 2: depression; 3: hopelessness; 4: self-depreciation; 5: guilty ideas of reference; 6: pathological guilt; 7: morning depression; 8: early awakening; 9: suicide; 10: observed depression

**Figure S5. Node strength stability in the case-dropping bootstrap procedure**

| **Low socioeconomic status** | **High socioeconomic status** |
| --- | --- |
| **** | **** |

**Figure S6. Edge weight accuracy estimated by bootstrapped confidence intervals**

| **Low socioeconomic status** | **High socioeconomic status** |
| --- | --- |
| **** | **** |

**Figure S7. Comparison by service engagement**

| **Subgroup** | **n** | **Mean SES (sd)** | **CS-coefficient edge** | **CS-coefficient node strength** |
| --- | --- | --- | --- | --- |
| Low SES | 453 | 19.19 (4.85) | 0.751 | 0.751 |
| High SES | 465 | 6.41 (3.69) | 0.673 | 0.751 |

CS-coefficients: maximum drop proportions to retain correlation of 0.7 in at least 95% of the samples


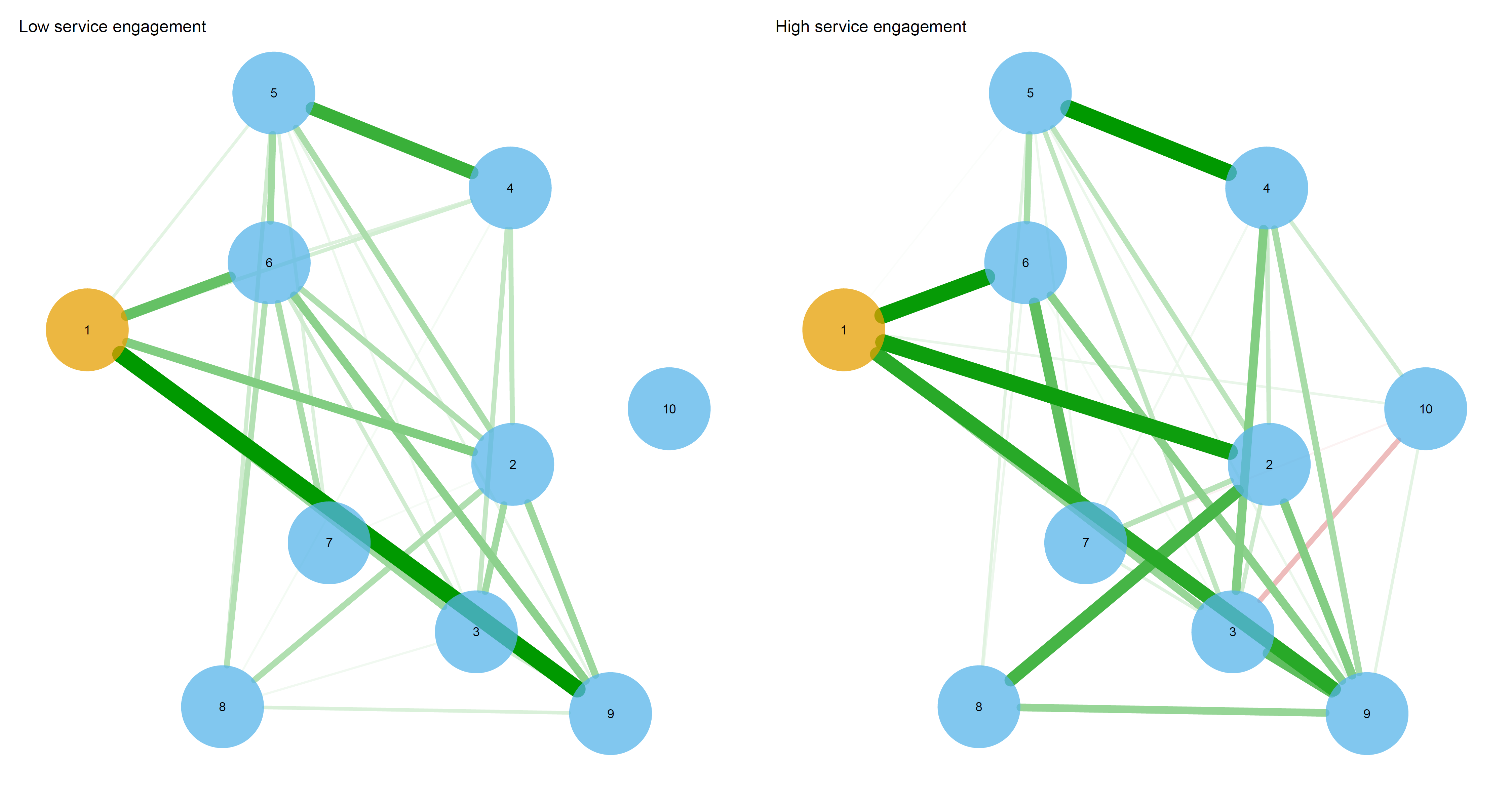


**Legend.** 1: PANSS G12 insight; 2: depression; 3: hopelessness; 4: self-depreciation; 5: guilty ideas of reference; 6: pathological guilt; 7: morning depression; 8: early awakening; 9: suicide; 10: observed depression

**Figure S8. Node strength stability in the case-dropping bootstrap procedure**

| **Low service engagement** | **High service engagement** |
| --- | --- |
| **** | **** |

**Figure S9. Edge-weight accuracy estimated by bootstrapped confidence intervals**

| **Low service engagement** | **High service engagement** |
| --- | --- |
| **** | **** |

**Figure S10. Comparison by illness severity (PANSS total score)**

| **Subgroup** | **n** | **Mean PANSS (sd)** | **CS-coefficient edge** | **CS-coefficient node strength** |
| --- | --- | --- | --- | --- |
| Low PANSS | 451 | 56.75 (11.09) | 0.594 | 0.672 |
| High PANSS | 467 | 93.50 (16.16) | 0.749 | 0.749 |

CS-coefficients: maximum drop proportions to retain correlation of 0.7 in at least 95% of the samples


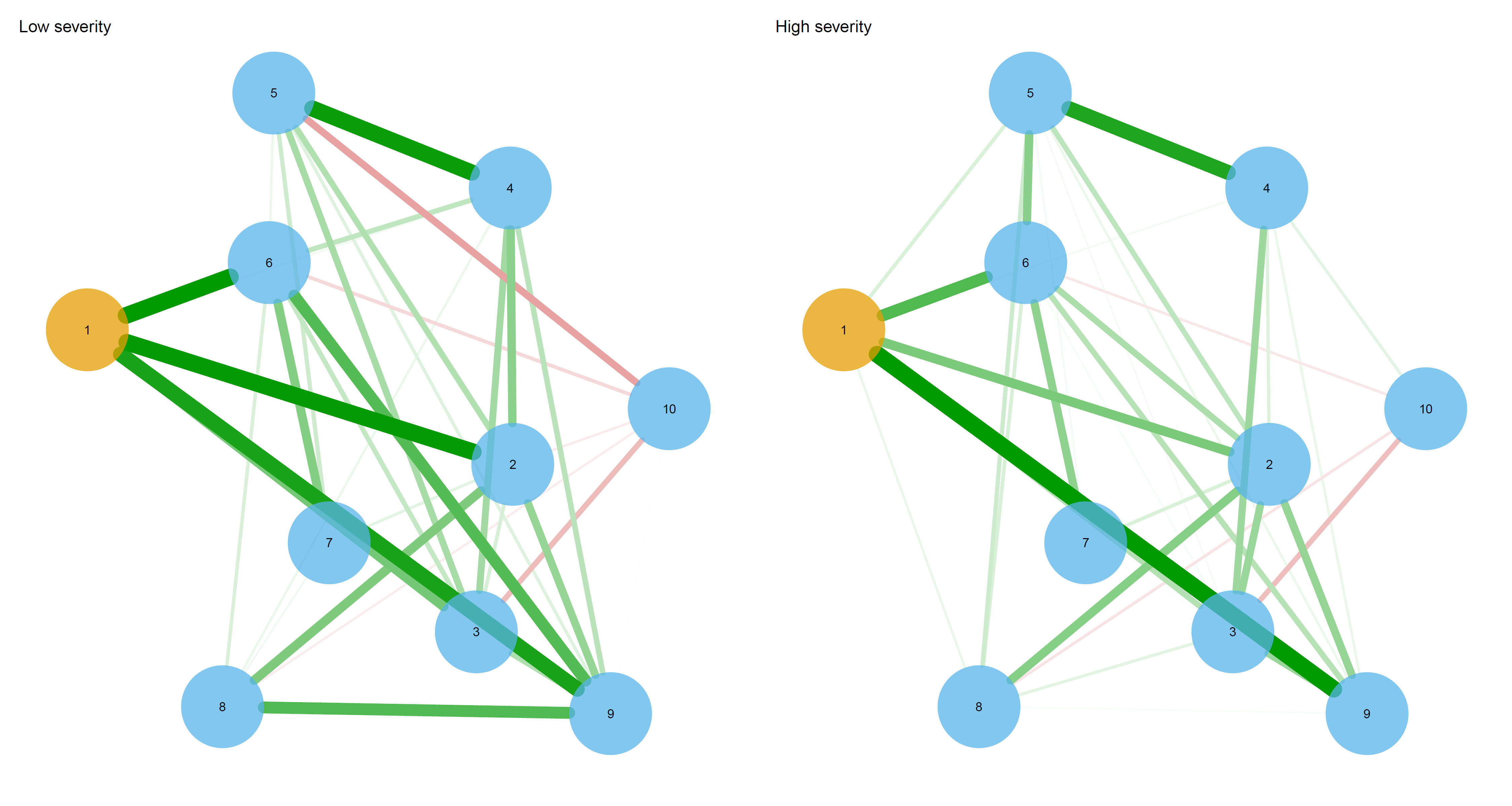


**Legend.** 1: PANSS G12 insight; 2: depression; 3: hopelessness; 4: self-depreciation; 5: guilty ideas of reference; 6: pathological guilt; 7: morning depression; 8: early awakening; 9: suicide; 10: observed depression

**Figure S11. Node strength stability in the case-dropping bootstrap procedure**

| **Low severity** | **High severity** |
| --- | --- |
| **** | **** |

**Figure S12. Edge-weight accuracy estimated by bootstrapped confidence intervals**

| **Low severity** | **High severity** |
| --- | --- |
| **** | **** |

**Figure S13. Strength centrality of the extended network**

**Figure S14. Node strength stability in the case-dropping bootstrap procedure**

| **n** | **CS-coefficient edge** | **CS-coefficient node strength** |
| --- | --- | --- |
| 855 | 0.891 | 0.854 |

CS-coefficients: maximum drop proportions to retain correlation of 0.7 in at least 95% of the samples

**Figure S15. Edge-weight accuracy of the extended network estimated by bootstrapped confidence intervals**


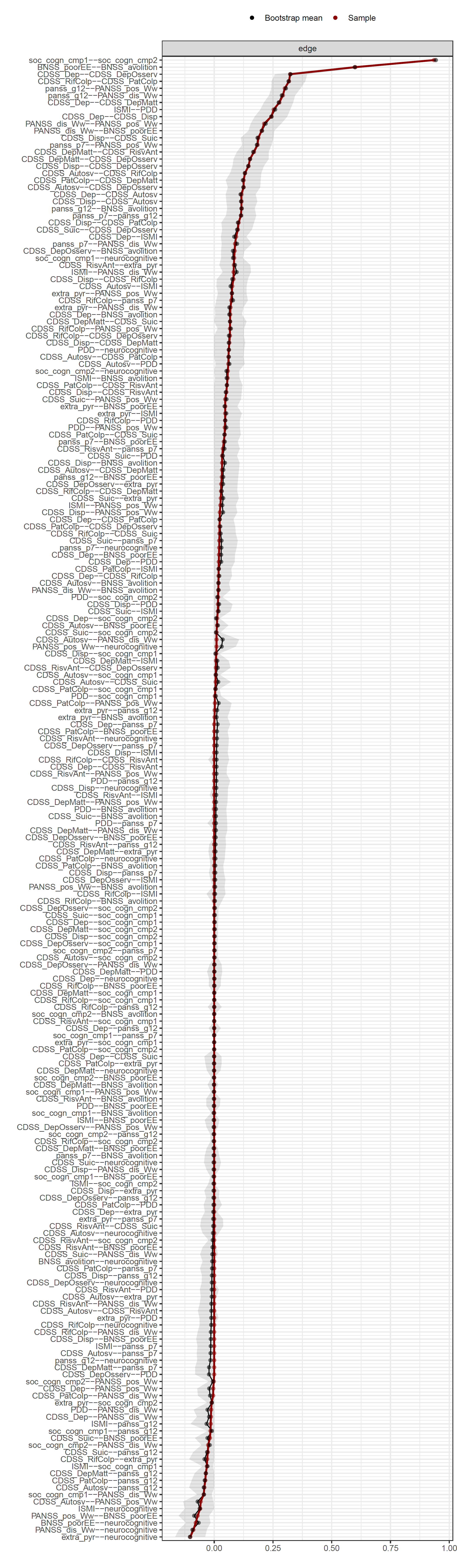


**Figure S16 DAG of depressive symptoms and insight, arc strength**

**Table S4. Arc strength and regression coefficients for the DAG of depressive symptoms and insight**

| **Edge** | **Arc strength** | **Regression coefficient** | **% of observed direction across bootstrap iterations** |
| --- | --- | --- | --- |
| G12_insight \| self_deprec | -0.21 | -0.04 | 0.93 |
| G12_insight \| guilty_id_refer | -6.91 | 0.06 | 0.89 |
| depression \| morning_depr | -57.70 | 0.34 | 0.87 |
| hopelessness \| self_deprec | -39.37 | 0.33 | 0.65 |
| hopelessness \| guilty_id_refer | -2.89 | 0.11 | 0.59 |
| hopelessness \| pathol_guilt | -37.07 | 0.28 | 0.54 |
| hopelessness \| early_wake | -2.10 | 0.10 | 0.70 |
| hopelessness \| suicide | -8.50 | 0.14 | 0.81 |
| hopelessness \| observ_depres | -166.63 | 0.49 | 0.65 |
| self_deprec \| guilty_id_refer | -10.80 | 0.16 | 0.51 |
| self_deprec \| pathol_guilt | -17.45 | 0.19 | 0.52 |
| pathol_guilt \| depression | -73.30 | 0.45 | 0.57 |
| pathol_guilt \| guilty_id_refer | -67.68 | 0.41 | 0.62 |
| pathol_guilt \| morning_depr | -10.65 | 0.14 | 0.59 |
| pathol_guilt \| early_wake | -0.03 | 0.09 | 0.74 |
| pathol_guilt \| suicide | -2.05 | 0.09 | 0.76 |
| morning_depr \| early_wake | -16.47 | 0.22 | 0.73 |
| morning_depr \| suicide | -0.07 | 0.08 | 0.52 |
| observ_depres \| self_deprec | -34.56 | 0.35 | 0.51 |
| observ_depres \| morning_depr | -12.54 | 0.19 | 0.66 |
| observ_depres \| suicide | -2.83 | 0.12 | 0.68 |
